# Chronic diseases of lifestyle curriculum: Students’ perceptions in primary health care settings

**DOI:** 10.4102/phcfm.v15i1.3775

**Published:** 2023-01-31

**Authors:** Sanet van Zyl, Willem H. Kruger, Corinna M. Walsh

**Affiliations:** 1 Department of Basic Medical Sciences, Faculty of Health Sciences, University of the Free State, Bloemfontein, South Africa; 2 Department of Community Health, Faculty of Health Sciences, University of the Free State, Bloemfontein, South Africa; 3 Department of Nutrition and Dietetics, Faculty of Health Sciences, University of the Free State, Bloemfontein, South Africa

**Keywords:** Chronic diseases of lifestyle, medical curriculum, community-based education, primary health care

## Abstract

**Background:**

Community-based primary health care (PHC) forms the foundation of healthcare in South Africa. Medical programmes need to equip future health practitioners to face the challenges of the rising burden of chronic diseases of lifestyle (CDL) in different communities. Community-based education (CBE) contributes to developing knowledge, skills and attitudes appropriate to the challenges experienced in the PHC context.

**Aim:**

To explore medical students’ perceptions of the current CDL curriculum and related programmes during CBE rotations.

**Setting:**

The study was conducted among fourth- and fifth-year medical students at the University of the Free State, South Africa.

**Methods:**

Focus group discussions were conducted and data were analysed thematically.

**Results:**

Themes included perceptions of the CDL curriculum, relevance thereof for the PHC setting and barriers and challenges to implementing PHC programmes. This study identified foundational CDL content that needs to be incorporated or revisited at strategic points. Participants identified the need to contextualise educational programmes and focus on affordable, culturally acceptable and holistic healthcare prevention strategies. Barriers and challenges included high patient load, resource constraints, the lack of continuous care and focus on communicable diseases. Community-based education rotations were described as meaningful opportunities to develop professional attributes, competencies and skills.

**Conclusion:**

This study identified foundational concepts to consider at key points throughout the curriculum. Incorporating creative and reflective learning activities in CDL modules can prepare students for the realities of PHC settings.

**Contribution:**

This study provides insight into medical students’ perceptions of the CDL curriculum and informs future curriculum content for CDL modules.

## Introduction

According to the World Health Organization (WHO), noncommunicable diseases are the leading cause of death globally. The projected increase in total deaths because of chronic diseases, from 36 million in 2018 to 55 million globally by 2030, will place a significant burden on healthcare systems, especially in developing countries.^1,2^

Chronic diseases constitute an essential part of the overall disease burden in South Africa, accounting for 51% of total deaths.^3^ Factors contributing to the growing tide of chronic diseases include epidemiologic transition (urbanisation), nutrition transition and socio-economic, cultural and behavioural or lifestyle-related risk factors.^4,5,6^ Modifiable lifestyle changes include adaptation to a more Westernised diet, physical inactivity, increased alcohol intake and smoking. These behavioural factors can lead to physiological and metabolic adaptations and the development of chronic diseases of lifestyle (CDL).^1,7,8^

The Global Action Plan for the Prevention and Control of Non-Communicable Diseases 2013–2020 called for policies and interventions to reduce premature deaths by 25% by 2025.^1,2,9^ Several health promotion strategies, guidelines and policies for communicable and chronic diseases were developed and implemented in the past decade. The 2030 vision of the South African National Development Plan is to prioritise nine long-term health goals, including reducing chronic diseases, improving the population’s health and well-being and strengthening the country’s health systems.^10^ The Strategic Plan for the Prevention and Control of Non-Communicable Diseases states the importance of strengthening community-based primary health care (PHC) and focusing on, *inter alia*, social and economic determinants of health, unhealthy lifestyles and metabolic risks to prevent chronic disease.^11^

Although some success in preventing and controlling chronic diseases has been reported, efforts to strengthen community-level prevention and control of chronic diseases in South Africa still need improvement.^12^ Barriers and challenges reported in PHC settings in South Africa range from complex health and nutrition transitions, shortage of healthcare workers, inadequate training of staff in the comprehensive care approach for chronic diseases, the lack of supervision, the lack of patient knowledge, awareness and self-management of chronic diseases.^13,14,15^

In response to the government’s commitment to improve the health profile of all South Africans and align with the health needs of local communities,^11^ South African universities introduced community-oriented educational approaches to PHC into their curricula. Since 2014, community-based education (CBE) has been incorporated into several modules throughout the 5-year medical curriculum offered at the University of the Free State (UFS). Furthermore, a CBE and Interprofessional Education (CBE–IPE) platform was established in the rural Free State town of Trompsburg in 2016. Since 2020, a structured CBE longitudinal approach was phased in to link student community projects in different modules and consolidate vertical integration in the curriculum.

Barss et al.^16^ emphasised the importance of evaluating the appropriateness of CDL curricula to ensure local and national future health practitioners are equipped to face national health priorities. Furthermore, the Health Professions Council of South Africa (HPCSA) encourages community-based research to advance healthcare development, inform teaching and learning practices in the undergraduate curriculum and assist in delivering health practitioners who can provide healthcare in context.^17^

The objectives of this study were to evaluate students’ perception of the current CDL curriculum and explore experiences related to CDL programmes during CBE rotations in PHC settings in the Free State.

## Methods

### Study design

This study followed a descriptive exploratory qualitative design to provide an in-depth understanding of medical students’ perceptions and experiences of the implementation of current CDL intervention programmes in Free State rural and urban settings.

### Study setting

This study was set in the context of the MBChB (undergraduate medical) curriculum offered at the Faculty of Health Sciences (FoHS), UFS, Free State. The 5-year medical programme comprises a preclinical (semesters 1–5) and clinical phase (semesters 6–10).

Theoretical CDL knowledge is incorporated in foundational (semesters 1–3) and system-based modules (semesters 4 and 5) offered in the preclinical phase and in the Internal Medicine and Family Medicine modules presented in the clinical phase of the programme.^18^ To apply theoretical knowledge in practice, a structured CBE longitudinal approach at various service-learning platforms of the FoHS was implemented in 2020. For example, students in the preclinical phase are exposed to the community and levels of healthcare in four modules presented in this phase. In the clinical phase, CBE rotations form part of the Family Medicine module. Fourth-year students (semesters 7 and 8) rotate in the rural town of Trompsburg in the Xhariep District and at primary health care facilities in the Botshabelo district during CBE–IPE training. Fifth-year students (semesters 9 and 10) complete CBE rotation in the urban Mangaung District at the University Community Partnership Programme (MUCPP) clinic.

### Study population and sampling strategy

The target population included all registered fourth- and fifth-year undergraduate medical students at the FoHs, UFS. The study sample comprised two groups. The first group was fourth-year medical students who had completed the required CBE rotation in the rural town of Trompsburg, and the second group was fifth-year students who had completed CBE rotation in the urban Mangaung district.

Focus group discussions were deemed an effective qualitative data-gathering method to present different perspectives and experiences and explore issues of concern.^19^ The aim was to recruit 8–12 participants per focus group. Brink et al.^20^ and Stalmeijer et al.^21^ suggest an optimal number of 8–10 participants, with a maximum of 12–15 participants per focus group.

This study followed a purposive sampling technique to recruit students with relevant CDL knowledge gained through theoretical sessions attended and experience gained during scheduled CBE rotations in order to provide their perspective and insight concerning the research topic.^22^ The study was done in the second semester of the academic year.

Recruitment included the electronic distribution of information letters (via class representatives) to all registered fourth and fifth-year students who had completed rotations in PHC areas. The information letter included the study’s purpose, inclusion criteria, what was expected during the focus group discussions and the voluntary nature of the study.^23^ Following a 3-week recruitment period, 22 students responded and the researcher scheduled an information session with the prospective participants. During this session, study information was verbally communicated, and participants had the opportunity to raise questions. After the researcher explained the voluntary nature of participation in the study (participants could withdraw at any time), written consent was obtained.^22^ All participants who gave written informed consent and fulfilled all the inclusion criteria were included in the study. The researcher used the inclusion criteria to ensure a heterogeneous sample including participants from both genders, different ethnicities and varied social backgrounds.

### Data collection

Focus group discussions were conducted in a seminar room in the Clinical Simulation and Skills Unit, FoHS. A set of questions was developed to guide the focus group discussions (Table 1). A senior member of the faculty who has a PhD degree in Health Sciences Education and experience as a facilitator for focus group discussions and nominal groups conducted the focus group discussions in English (the language of instruction). He is involved in the CBE–IPE training programme presented in the fourth year of the clinical phase of the medical programme. Focus group discussions lasted between 60 and 90 min. The facilitator followed an agenda compiled by the researcher that included an outline of the study purpose and a discussion of ground rules, confidentiality and respect for privacy before starting each focus group discussion. Each participant’s assigned number (identifier) was displayed on a tag, and a seating chart was used for note-taking purposes.^22^

**TABLE 1:** Focus group discussion guide.

Question number	Question
Question 1	‘What is your opinion about the information provided in the MBChB programme regarding overweight, obesity and the associated risk factors for the following chronic diseases of lifestyle: hypertension and diabetes?’
Question 2	‘What is your opinion about the information provided in the MBChB programme regarding interventions for chronic diseases of lifestyle (with specific reference to overweight, obesity, hypertension, diabetes) and the implementation thereof in a primary healthcare setting?’
Question 3	‘Which operational protocols or guidelines to address risk factors for chronic diseases of lifestyle were displayed at the primary healthcare facility?’
Question 4	‘Which areas of good practice, with reference to the effective implementation of CDL prevention and intervention programmes, did you observe during your CBE rotation in the primary healthcare facility?’
Question 5	‘Which prevention and intervention programmes observed in the primary healthcare facility integrated shared risk factors for lifestyle diseases (unhealthy diet, physical inactivity, smoking)?’
Question 6	‘What is your opinion regarding adequate opportunity to understand and experience acute management of chronic diseases of lifestyle (e.g. hypoglycaemia in a diabetic patient)?’
Question 7	‘What is your opinion regarding adequate opportunity to understand and experience ongoing management of chronic diseases of lifestyle (e.g. identify chronic complications in a patient with hypertension)?’

CBE, community-based education; CDL, chronic diseases of lifestyle; MBChB, Bachelor of Medicine and Bachelor of Surgery.

The focus group discussions were recorded with a digital recorder, while the principal investigator observed the sessions. Documenting focus group discussions comprised MP3 audiotape recordings and written field notes taken by the principal investigator. Immediately after each focus group discussion, the principal investigator conducted a debriefing session with the facilitator to note important nonverbal communication, group dynamics, the effectiveness of questions and new topics that emerged during the discussion.^22^

Because of the clear themes that emerged from the narratives as well as the saturation of themes, the researchers decided that the two focus group discussions held were sufficient for this exploratory study.^24,25^

### Data analysis

The principal investigator transcribed the recorded audio data verbatim and made field notes on the transcript, before listening to the audio recordings again and rereading the transcript to confirm accuracy.^23^ Following an inductive strategy, the thematic analysis included data coding, identifying categories and subcategories and recognising emerging themes using Microsoft Office software (e.g. Microsoft Word and Excel) (Microsoft Corporation, Redmond, Washington, United States).^21^

A systematic approach, adapted from Braun and Clark,^26^ was followed to organise and interpret the qualitative data. The principal investigator first read through the transcript to obtain a general overview of the data. Statements relevant to the research topic and information not related to the research topic were separated (step 1). The researcher again reviewed the transcript notes, breaking the data into words, phrases and sections of text that reflected the participants’ specific thoughts. The relevant text was highlighted in a unique colour to correspond with each code represented (step 2). The researcher then identified recurring codes (categories) for individuals, within a group and among different focus groups. The categories were grouped into themes and subthemes (step 3). After step 3 was completed, the main supervisor (co-author), an expert in qualitative research approaches, and the co-supervisor (co-author), both senior faculty members, moderated the processes independently by reviewing and comparing the codes and identified themes and subthemes against the dataset to ensure the credibility of the process (step 4). The authors then discussed, amended and finalised the themes and subthemes. The agreed-upon themes were then connected to develop descriptive or overarching themes that relate specifically to CDL and CDL intervention programmes as seen through the participants’ eyes (step 5).^24,25^ Credibility, transferability, dependability and confirmability were used as criteria to ensure the trustworthiness of this study.^20^ Credibility was ensured by using the most appropriate methods for data collection and recruiting medical students who had relevant experience implementing CDL intervention programmes. The co-authors moderated the thematic exploration to ensure completeness and validity of the results. This was to confirm that categories and themes covered the data and that relevant data had not been accidentally excluded or irrelevant data included.^20,27^ The principal investigator used a ‘thick’, detailed description of the study context to enable other researchers to conduct a similar study in other settings.^27^ Dependability was assured by an audit trail, including detailed documentation of the chronology of the methodological and data analysis process through which the findings were derived. Reflexivity was ensured by recognising and limiting the researcher’s bias because of background or past experience. The researcher is a healthcare professional involved in the training of medical students in the preclinical years and a coordinator for the second and third academic year of the medical programme; moreover, the researcher is also involved in the curriculum review and renewal processes. This was done by intentionally excluding preconceived ideas and subjective opinion through reflective thinking and adhering to stringent ethical processes.^21,28^ Furthermore, after the researcher completed the systematic process to organise and explore associations between themes, the internal consistency of the interpretation of data and thematic saturation were confirmed by the other two authors.

### Ethical considerations

Ethics approval was obtained from the Health Sciences Research Ethics Committee of the UFS (UFS-HSD2017/1435), the Free State Department of Health and local municipalities. The principal investigator (with the translator’s assistance) explained the voluntary nature of the study before obtaining written informed consent from all participants.

## Results

A total of 22 participants (18 women and 4 men) participated in focus group discussions; 12 fourth-year MBChB students and 10 fifth-year MBChB students. Table 2 provides an overview of the overarching themes and concepts, main themes and subthemes that emerged during the analysis. The discussions about the five main themes and associated subthemes are followed by quotes reflecting the students’ perceptions and experiences.

**TABLE 2:** Overarching concepts, global themes and subthemes identified from the focus group discussions.

Overarching themes and concepts	Main theme	Subthemes
Academic knowledge of chronic diseases of lifestyle	1. Responsiveness and relevance of the MBChB (undergraduate medical) curriculum to the growing burden of CDL	1.1 Quantity of CDL curriculum content 1.2 Quality of CDL curriculum content 1.3 Transition from theory to clinical practice
	2. Realities faced in PHC settings	2.1 Alignment of theoretical content with the health needs of the community 2.2 Implementing healthcare in context
Experiences of chronic diseases of lifestyle programmes in rural and urban PHC settings	3. Implementation of CDL programmes in resource-constrained PHC settings	3.1 Health promotion and education programmes: a need to contextualise health needs and offer culturally acceptable and holistic healthcare 3.2 Effectiveness of existing prevention and intervention programmes
	4. Acute and ongoing management of CDL	4.1 Challenges and barriers observed for the management of acute complications of CDL 4.2 Challenges and barriers observed for the ongoing management of CDL 4.3 Areas of good practice observed in the management of CDL
Development of professional attributes and competencies	5. Reflection on the CBE experience	5.1 Meaningful experiences that broaden the understanding of PHC and the concept of delivering healthcare in context 5.2 Opportunity to develop graduate competencies 5.3 Improve the understanding of the social-cultural context of health and disease in different communities 5.4 Psychological impact of the CBE experience

CBE, community-based education; CDL, chronic diseases of lifestyle; PHC, primary health care.

*Themes 1 and 2* focus on participants’ academic knowledge, including their perception of the CDL curriculum content, the relevance for different PHC settings and the community’s health needs. *Themes 3–4* reveal the participant’s perceptions, observations and experiences relating to the functioning of CDL programmes in the PHC settings. *Theme 5* focuses on participants’ CBE experience and the opportunities to develop professional attributes, competencies and skills. Statements from fourth-year MBChB students are referred to as MSG1 (medical student group one), followed by the participant’s number. Statements from fifth-year MBChB students are referred to as MSG2 (medical student group two), followed by the participant’s number.

### Theme 1: Responsiveness and relevance of the MBChB curriculum to the growing burden of chronic diseases of lifestyle

Reflecting on the *CDL curriculum content*, most participants of both groups reported satisfaction with the *quantity of information* provided in the curriculum and the broad spectrum of CDL discussed. Participants agreed that the topics addressed in the preclinical phase were relevant and representative of what they observed during CBE rotations in the different PHC settings:

‘You don’t realise how real it is until you get a patient right in front of you, and then you see everything that you were learning about. I’m always really impressed when you get a patient, how it is really typical of what you have learned about in class.’ (MSG1, No. 3, female, 22 years)

‘Yes, I remember we were in a group with other disciplines, the OTs [*occupational therapists*], nurses, and staff. We were the ones who knew everything about all the chronic diseases, diabetes, hypertension, … so we were very confident.’ (MSG2, No. 10, female, 23 years)

Regarding the *quality of the information*, participants felt that the information conveyed, specifically epidemiology and treatment management, was not relevant for the South African setting. Some participants identified that new and fast-developing fields, for example, epigenetics, were omitted, while others felt that some content was obsolete:

‘My information for hypertension and diabetes comes from Davidsons [*prescribed textbook*], but the epidemiology, you don’t get epidemiology or stats for this country.’ (MSG1, No. 1, female, 22 years)

‘We can focus on the latest research regarding epigenetics; things like that is [*sic*] very actual at the moment, hot topics.’ (MSG1, No. 4, female, 22 years)

‘We don’t have a uniquely South African syllabus on healthy lifestyle modification in South Africa.’ (MSG1, No. 10, male, 22 years)

‘In pharmacology, we studied everything, and we don’t see any of that, so the non-practical drugs. They [*pharmacology lecturers*] could just highlight this is what we use in South Africa.’ (MSG2, No. 10, female, 23 years)

Participants referred to the *transition from theory to practice* and the gap between theoretical knowledge obtained in the preclinical phase and practical application in the clinical phase:

‘We do need the foundation of the basic medical sciences. I feel there was just too much information. In the clinical phase; we were expected to know automatically exactly how everything is supposed to be. I feel there was a gap between the theory and the practical application.’ (MSG2, No. 1, female, 24 years)

‘It is important to improve primary health care, that we don’t see so many complications. We are being taught that, but just a reinforcement of primary and preventative measures can be improved and be a lot better.’ (MSG2, No. 8, female, 23 years)

### Theme 2: Realities of primary health care settings

Participants commented on the discrepancy between *the theoretical knowledge obtained in CDL programmes and the realities of implementing these programmes in PHC settings*:

‘The problem is they [*lecturers*] tell us you have to have a proper diet and you tell your patients to have a proper diet, and you know a proper diet [*consists*] of some meat, some veggies, but in the South Africa setting, it is really impossible to afford that.’ (MSG1, No. 5, male, 22 years)

‘They [*lecturers*] also taught us the importance of screening for chronic lifestyle disease. The only problem with that, especially in our rural clinics, there is no equipment to screen the patients, while I think that is very important in preventing a lifestyle disease before it begins to escalate.’ (MSG1, No. 6, female, 22 years)

‘There are no protocols, especially in the rural areas, as what to do with a patient when you find, for example, high glucose at a lifestyle club. We learn about these treatments, but it is very rare that the treatment is available.’ (MSG1, No. 11, female, 22 years)

The importance of *teaching and implementing healthcare in context*, for example, the need for affordable, culturally acceptable and holistic healthcare, is demonstrated in the following:

‘They tell a person to eat healthy, but they don’t consider the person’s background. If you say the person [*patient*] has to eat healthy, and they don’t have food, but you don’t teach them how to make a [*vegetable*] garden. We have to individualise the patients, and I don’t think they emphasised that much.’ (MSG1, No. 3, female, 22 years)

‘There was a nice diabetic poster put up by the students in the clinic, and everyone said it is completely useless because it is in a language that they [*patients*] do not understand. It is not applicable to their specific diet or culture. We should have been taught some of those things, what food is available, the cultural norms and teaching us conversational language.’ (MSG2, No. 4, female, 23 years)

### Theme 3: Implementation of chronic diseases of lifestyle programmes in resource-constrained primary health care settings

Participants acknowledged that visiting PHC facilities and patients’ homes during CBE rotations increased their understanding of healthcare systems and the challenges experienced in the different PHC settings. Participants indicated several barriers and challenges to the effective implementation of *health promotion and educational strategies* for CDL: a lack of educational material, outdated information on educational material, poster displays not optimal, language barriers and posters that focus primarily on communicable diseases (e.g. human immunodeficiency virus [HIV] and tuberculosis [TB]):

‘Diabetes posters were in the clinics, but I have not seen any obesity posters or healthy diet poster. You see dietetics things about eating salmon; once again, it is way out of context.’ (MSG1, No. 5, male, 22 years)

‘They [*lecturers*] teach us a lot about the risk factors for developing chronic lifestyle diseases, but I don’t think they emphasised it enough in the community outside of the healthcare centre. I think this is a problem in the community in terms of promoting a better and a healthier lifestyle because that is where it all starts, and I don’t think they [*staff at the primary care facility*] know how important this is.’ (MSG1, No. 6, female, 22 years)

‘Sometimes the posters are only in English.’ (MSG2, No. 5, female, 23 years)

Participants accentuated the practical contributions they can make in *promoting health education* during CBE rotations in PHC:

‘When we did our presentation, we did it on the diabetic foot. We would explain from the pictures; there was very little wording. You could see that they [*patients*] understood more. We even showed them instead of just telling them, “this is how you wash your foot”.’ (MSG1, No. 3, female, 22 years)

Participants noted that effective CDL prevention strategies and protocols were followed for communicable diseases (e.g. HIV and TB, immunisation and needle-stick injury) in both urban and rural PHC settings, while this was *less evident for CDL*:

‘I have seen a lot of TB posters in MUCPP [*Mangaung University Community Partnership Programme clinic*], but I have never seen anything about hypertension anywhere.’ (MSG2, No. 7, female, 24 years)

Challenges and barriers observed for the effective implementation of *CDL prevention programmes* include a lack of medication and resources for screening, high patient load and staff shortages:

‘The load on the staff is too much, so there is no time to explain everything; you just want to get through the line [*of patients*].’ (MSG1, No. 4, female, 22 years)

‘I think it is quite lacking due to not having the equipment, not having the manpower to basically see all patients and serve the whole community.’ (MSG1, No. 7, female, 22 years)

The WHO^9^ defined integration of health services as the:

[*M*]anagement and delivery of health services so that clients receive a continuum of preventive and curative services, according to their needs over time and across different levels of the health system. (pages 20–21)

Asked about the integration of shared risk observed in the different PHC settings, participants confirmed the lack of integration to the challenges experienced (e.g. staff shortages, time constraints):

‘The mom is maybe obese, and she has all [*these*] risk factors, but due to time, we don’t take that extra five or ten minutes and just quickly check the blood pressure, do a glucose screen and just eliminate risk factors before she even gets the disease.’ (MSG1, No. 4, female, 22 years)

‘I think it was at a diabetes, hypertension clinic, the patients [*were*] supposed to have yearly follow-up where blood should be taken, it should be arranged to check their eyes. With some of the patients I could not find one [*follow-up form*] in the file. I look for results, and the last was done in 2012, and they should be done every year.’ (MSG2, No. 3, female, 24 years)

### Theme 4: Acute and ongoing management of chronic diseases of lifestyle: Challenges and successes

Participants mentioned that the lack of resources in PHC clinics contributed to the difficulties observed in the acute management of CDL on PHC level:

‘Also, I think what might help in our context is to be taught what every level of health care’s role is in these lifestyle diseases because we were not really sure.’ (MSG1, No. 10, male, 22 years)

‘I saw a patient in the clinic at Springfontein that has diabetes and hypertension, and heart problems, and she came in with signs and symptoms of heart failure, but there was [*sic*] no ECG stickers in the clinic; I think that is a big problem.’ (MSG2, No. 7, female, 24 years)

Regarding the ongoing management of CDL, poor control of CDL contributed to lack of patient education and compliance, staff and resource shortages, high patient load (especially in the urban setting), late presentation because of inadequate risk prevention measures, lack of continuous care (incomplete files, different physicians), treatment management problems and cultural and traditional beliefs. Participants mentioned that the focus in the PHC setting remains mainly on treatment regimens and not customised preventive measures:

‘It is very important that they customise the interventions for a South African setting.’ (MSG1, No. 9, female, 22 years)

‘In Trompsburg, we checked each patient’s blood pressure and medication. If the blood pressure was too high, we adjusted the medication, but you don’t check the patient’s weight; if the patient is losing some weight or if the BMI is above 40.’ (MSG1, No. 3, female, 22 years)

‘I just want to say a few things about intervention; the medication patients get, they don’t know why they have these different medications. I think then they don’t use it as it is prescribed because they don’t really think it is necessary.’ (MSG1, No. 4, female, 22 years)

‘Regarding the interventions, I think it is quite lacking due to not having the equipment, the manpower to basically see all those patients and serve the whole community.’ (MSG1, No. 7, female, 22 years)

‘The chronic management of our patients is very difficult in our clinics because our patients see different doctors the whole time.’ (MSG2, No. 2, female, 23 years)

‘Patients have these carry cards where they [*clinic staff*] write their blood glucose levels or blood pressure levels, but sometimes you find that their cards are incomplete. What are the point of having these things if you are not going to be consistent and fill it in every time.’ (MSG2, No. 7, female, 24 years)

*Successful practices* include active participation in lifestyle groups in the rural area and effective screening practices at local schools, while successful screening practices were observed at the diabetes clinic located at a primary-level hospital in the urban area:

‘In terms of ongoing management, I feel like those lifestyle group therapy [*rural setting*] are aimed in terms of that. They [*clinic staff*] identify the hypertensive or diabetic patients, and they make sure that every week they come together and, let’s say one of the patients this week suffered from some sort of problem, they discuss it so that everyone gets educated together.’ (MSG1, No. 2, female, 21 years)

‘If we speak about Trompsburg, there is a very good screening programme for the school children. I think that would be amazing if they could implement it in the country on a national level …’ (MSG1, No. 5, male, 22 years)

‘I think in the small communities [*rural setting*], there is a good interpersonal relationship that has been built between the healthcare workers and the community.’ (MSG1, No. 11, female, 22 years)

‘When we were rotated at policlinic B at the diabetic clinic [*urban setting*], each patient will come in, and their blood pressure is taken, the finger prick glucose, dipstick is done, they do the Snellen chart, so in some clinics, there is some intervention that is working.’ (MSG2, No. 5, female, 23 years)

### Theme 5: Reflection on the community-based education experience

Participants agreed that CBE was a *meaningful experience and assisted in broadening their understanding of PHC and the concept of delivering healthcare* in context. Participants stressed the importance of mutual benefit for patients and students and emphasised that they want to play an active role in CDL prevention and intervention strategies, such as health dialogue, education and other strategies:

‘In Trompsburg, they have a diabetes club every week. We started by checking the patients’ blood pressure and glucose levels. It is a great way of using students to help with the screening load of the patients, and we gave them information, especially on the risk factors which cause diabetes.’ (MSG1, No. 6, female, 22 years)

‘CBE was very helpful to us, and I learned that we must be examples to our patients. We live in communities with our patients, and they look at us, our lifestyle.’ (MSG2, No. 2, female, 23 years)

‘It was not just about the patient who came to us for help. We also showed the patients that we care for them and went into their homes to find out the challenges they face with regards to their illnesses.’ (MSG2, No. 2, female, 23 years)

‘We saw a patient with critical limb ischemia, we taught them how to take care for their limbs. I think that was a good intervention.’ (MSG2, No. 6, female, 25 years)

‘CBE is very helpful. It helps for us to see where it starts, and it is important for us to improve primary health care, that we don’t see so many patients with complications.’ (MSG2, No. 8, female, 23 years)

The HPCSA^17^ refers to core competencies that should be developed during the training of undergraduate medical students. For example, graduates must develop a *range of competencies and essential skills* relevant to the PHC setting. Participants indicated that CBE and IPE provided the opportunity to develop communication and collaboration skills and the different roles expected of a healthcare provider (e.g. being a health promoter and health advocate). Regarding collaboration and delivering integrated, holistic care, participants believed that earlier exposure (e.g. receiving lectures from the multidisciplinary team members from the first year in the preclinical years before CBE rotation) would be more beneficial:

‘I don’t feel that we are adequately trained, specifically regarding dietetics and when to refer accordingly.’ (MSG1, No. 1, female, 22 years)

‘I think we had maybe one lecture in the first year from dietetics, no lectures from physiotherapy, none from occupational therapy and maybe one or two from nursing. We could learn a lot more about when to refer and what they can help us help the patient with.’ (MSG1, No. 10, male, 22 years)

‘I think it would be helpful if we could maybe have lecturers from, say, occupational therapist and physiotherapist within each rotation that we have a broader vision of how they are involved.’ (MSG1, No. 11, female, 22 years)

The WHO^1^ emphasised the importance of considering social determinants of CDL throughout the patients’ lives. Participants reported that CBE provided them with an opportunity to observe the *social, cultural and economic contexts of health and disease* in communities and the role that they, as future physicians, and the patient (self-care), can play:

‘We focus more on diabetes, hypertension, but we don’t really focus on the holistic individual as a person; we focus more on to restrict your salt intake, but what happens if you are depressed, suicidal, burnout, or if stress is causing you your illnesses?’ (MSG1, No. 12, female, 24 years)

‘I feel that is something that surely [*lacks*] in that rotation. We should have been taught what food is available to the patients, what are the cultural norms – teaching us conversational language so that you can get ideas across to your patient, without having to abandon the conversation.’ (MSG2, No. 4, female, 23 years)

One recurring theme emerging from the focus group discussions was the psychological effect of CBE exposure. Participants reported feeling anxious and stressed during CBE rotations, for example, ‘not feel comfortable, it was a stress factor, distressing, you are a bit worried, you are not sure, feel rather powerless’. Contributing factors to the emotions experienced included lack of supervision, language barriers, resource constraints and protocols not followed or not available in PHC settings:

‘There are no protocols, especially in the rural areas, as what to do with a patient when you find, for example, a high glucose at a lifestyle club. And it [*makes*] it specifically difficult if the facilitator is not with you. In one specific instance, we had a lifestyle group where we were there, the doctor was not with us, and we could not get hold of the doctor or the programme facilitator in the region, and it’s very distressing if you don’t know what to do and you have identified a problem.’ (MSG1, No. 11, female, 22 years)

Additional factors such as the lack of knowledge of CDL acute management, different levels of referral and the roles and responsibilities of healthcare workers when patients need to be referred for special care contributed to the psychological stress experienced by the participants:

‘They don’t tell us who to call when you have [*a patient with*] high blood pressure or blood sugar, or if it is so severe, phone the ambulance; here is the ambulance’s number. They don’t tell us which services will be given to us in acute situations, especially in the rural clinic.’ (MSG1, No. 1, female, 22 years)

‘When we were in Trompsburg, I realise for the first time how important the referral system.’ (MSG1, No. 6, female, 22 years)

‘I remember we were at a clinic; this lady had a DVT [*deep vein thrombosis*]. We all knew what to do as medical students. I don’t know if there was a referral problem or a lack of insight from the staff side.’ (MSG2, No. 10, female, 23 years)

## Discussion

This study confirmed that CBE is a valuable experience in medical students’ teaching and learning process. The students emphasised the importance of contextualising educational programmes in these communities and focusing on affordable, culturally acceptable and holistic healthcare strategies for CDL. Furthermore, this study identified foundational CDL content that needs to be incorporated or revisited at strategic points throughout the curriculum. Students identified specific challenges to the effective implementation of CDL programmes in urban and rural PHC settings. These challenges are not unique, as other African countries are experiencing similar challenges,^29,30^ but such challenges should not be ignored. Research on students’ experiences is necessary as the observations during CBE rotations can inform teaching and learning practices.

The integrated curriculum provides an important way to close the gap between theory and practice by reinforcing and integrating basic and clinical sciences.^31^ Challenges often experienced in integrated curricula include making meaningful connections between the theoretical and applied content and ensuring a smooth transition from theory to practice.^32^ In this study, participants identified such challenges by highlighting a need to revisit CDL protocols, prevention and intervention programmes before CBE rotation in the clinical phase commences. Participants also identified a disconnect regarding implementation of management protocols, where references were often made to CDL management as applicable for a tertiary or private healthcare setting and not what transpired in PHC settings. Gouda et al.^33^ commented on the importance of incorporating preventive and lifestyle interventions in CDL curricula to equip students for PHC settings. Refresher courses in the clinical phase, before CBE–IPE rotations commence, can ensure a smooth transition from theory to practice and equip students for the realities faced in PHC settings. These courses should focus on preventive lifestyle interventions and treatment management protocols (e.g. essential drugs available in the public sector), roles and responsibilities of the PHC team and referral pathways). Reporting on global progress, the Commission on the Social Determinants of Health^34^ emphasised the commitment and efforts of many countries at the national and local levels to improve the social determinants of health, quality and effectiveness of medical care. Although South Africa’s National Health Promotion Policy and Strategy (2015–2019)^35^ focuses on creating an enabling environment and strengthening human resource capacity to deliver health promotion services, there are still challenges and barriers to CDL health promotion and intervention strategies. Parker et al.^36^ identified a lack of resources (including staff and equipment), high patient load and noncompliance as challenges and barriers experienced at PHC level for the effective implementation of CDL programmes. Participants in this study also observed staff shortages (urban and rural setting) and resource constrains (rural setting) and the high patient load (especially in the urban setting). Additionally, participants in the current study reported the lack of continuous care (incomplete files, different physicians in the rural setting), interventions not relevant for the socio-economic context (urban and rural setting), late presentation because of inadequate risk prevention measures (in the rural setting), cultural and traditional beliefs and greater emphasis on infectious diseases (HIV, TB). The latter was also observed in another South African community-based study. Madela et al.^37^ commented on the strong emphasis on communicable diseases at the PHC level while observing deficiencies in CDL education, care and management. Furthermore, participants in the current study reported the lack of patient education, outdated educational material, posters that focus mainly on communicable diseases and health education that is not delivered in context (urban and rural setting). Chen et al.^38^ suggested that intervention strategies should include the design of culturally sensitive programmes and educational materials to improve patients’ knowledge and self-management.

Health empowerment focuses on strengthening the roles of individuals or communities to manage their own health by adopting healthier habits and providing health education and support to manage their disease.^39^ Participants in this study reported that the focus in the primary setting remains mainly on treatment regimens and not preventive measures. However, in the current study, some successful health empowering practices in the rural setting included fruitful health dialogue, support for hypertension and diabetes patients in lifestyle groups and a point reward system to enhance patient compliance. Sheik et al.^40^ also reported on the effectiveness of chronic disease lifestyle groups in the Western Cape, South Africa.

Medical graduates must demonstrate the development of various competencies and essential skills relevant to the PHC setting. These include preventive, promotive and therapeutic interventions, practising effective communication as a core clinical skill and timely consultation and collaboration with other healthcare professionals.^17^ This study confirmed that CBE and IPE were meaningful experiences that can assist students in contextualising and broadening their understanding of communities’ health needs and developing collaboration and communication skills. Frenk et al.^5^ emphasised that the modern curriculum must enhance interprofessional training, break down divides between different professions and develop generic competencies, such as communication, leadership and management skills. Although CBE and IPE enhanced the development of competencies and skills necessary for collaborative practice, participants in the current study felt that earlier exposure, for example, to interprofessional teaching and learning from the first year in the preclinical curricula, would be beneficial.

The biopsychosocial model of health refers to the holistic, integrated approach that includes biological, psychological and social determinants (economic, environmental and cultural) that contribute to the development of diseases.^41,42^ Although participants’ comments on the social determinants of health affecting different communities demonstrated social responsiveness, learning opportunities need to be created in the preclinical CDL curriculum for students to develop social, cultural and self-awareness.

Participants in this study referred to the critical role that students can play in patient education and related self-management and self-care. Health empowerment provides opportunities and skills to enable individuals or communities to take control of their own health needs.^39^ In a collective effort between higher education institutes and the Department of Health in Ireland, an undergraduate chronic disease curriculum was developed that incorporated content on patient self-management and self-care. This assisted the students as future healthcare practitioners in developing competencies and skills to advise patients on self-management and assist patients in becoming actively involved in their own healthcare.^43^

### Study limitations

This study was conducted at only one higher education institution in South Africa, which included only two service-learning sites – one urban and one rural – because of resource constraints. Furthermore, the study was conducted in one province in South Africa and represents specific challenges experienced within the particular province in an urban and rural PHC context. The researchers also acknowledge the small sample size. However, as a quality criterion in qualitative research, the transferability of the findings to different settings is enhanced through a thick description of findings in context and resonating with available literature in various settings.^44^

### Recommendations

The findings of this study led to the following recommendations regarding learning in CDL modules.

The CDL learning modules in the medical curriculum must provide creative learning opportunities for students to enhance their contextual understanding of the multifactorial aetiology that drives the CDL disease processes in communities. Incorporating reflective activities as part of a student professional development e-portfolio throughout the curriculum can enhance students’ sociocultural and self-awareness. Furthermore, students can be involved in developing healthy lifestyle and health empowerment information or educational material during the CBE component of the CDL curriculum. The early exposure to interprofessional teaching and learning in the preclinical years of study is advised, as it can enhance the students’ understanding of the role that different health professionals play in the holistic care of patients with CDL. Additionally, a treatment management refresher course before the CBE–IPE rotations focusing on the core knowledge applicable to the PHC setting can add value, for example, a course on the use of the WHO’s ‘package of essential non-communicable (PEN) disease interventions for primary health care’ (p. 67).^45^ Because CDL is not unique to the Free State, it is also advised that a national curriculum for CDL be developed through a collaborative effort between educationalists and policymakers, to produce healthcare professionals sensitive to the health needs, physical, mental and social well-being of communities, which can ultimately contribute to a healthy life for all.

## Conclusion

The escalating burden of CDL in rural and urban communities in South Africa necessitates the need for effective community-based health promotion and care. Community-based education should allow students to apply academic knowledge in context and observe the challenges faced during the implementation of CDL programmes in resource-constrained PHC settings. Participants in this study identified specific challenges in the curriculum with particular reference to CDL content and identified important foundational knowledge that needs to be incorporated or revisited at strategic points throughout the curriculum. Although CBE provided a valuable opportunity to develop the student’s knowledge, skills and attitude, this study revealed that more emphasis should be placed on the students’ understanding of the biopsychosocial determinants of CDL and the development of self-efficiency (knowledge and confidence) to prepare future physicians for the realities of rural and urban PHC settings.
